# Bifurcation analysis of anti-phase oscillations and synchrony in the tadpole central pattern generator

**DOI:** 10.1186/1471-2202-15-S1-P94

**Published:** 2014-07-21

**Authors:** Roman Borisyuk, Robert Merrison-Hort

**Affiliations:** 1 School of Computing & Mathematics, Plymouth University, Plymouth, Devon, PL4 8AA, UK

## 

Recent experimental findings in the laboratory of Wen-Chang Li (St. Andrews University, UK) demonstrate that the same neural Central Pattern Generator (CPG) within the hindbrain/spinal cord of the *Xenopus* tadpole can produce both reliable anti-phase oscillations between the left and right sides of the body (swimming) and long bouts of in-phase synchronous activity. The key element of the CPG circuit includes a pair of neurons on each side of the body at about the same position: an excitatory descending interneuron (dIN) and an inhibitory commissural interneuron (cIN). A simplified description of the CPG activity in swimming regime follows from experimental facts: dIN spiking on one side leads to excitation of the cIN on the same side, which inhibits the dIN on the opposite side causing a spike due to post-inhibitory rebound (PIR). Similarly, experiments suggest that synchrony appears as a result of simultaneous firing of dINs on both sides which leads to simultaneous firing of cINs on both sides, followed by inhibition of dINs (on opposite sides) and a new cycle of simultaneous dIN firing due to PIR.

**Figure 1:**
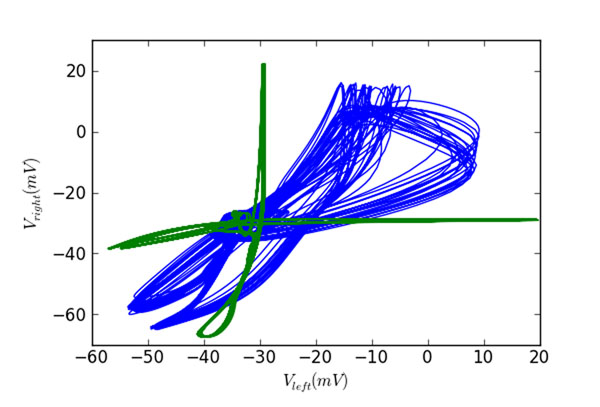
Phase portraits of the system during anti-phase (green) and in-phase (blue) activity. Horizontal and vertical axes correspond to membrane potentials of dINs at similar positions on the left and right body side respectively.

To study these exciting experimental findings we use biologically realistic anatomical and functional computational models [1-3]. The tadpole swimming model represents a 1.5 mm section of tadpole spinal cord with ~ 1,500 neurons, interconnected by ~ 85,000 synapses. Neuron dynamics are simulated according to a conductance based model of the Hodgkin-Huxley type. Additionally, gap junctions between dINs within 100µm of each other in the rostro-caudal direction are included. From studying the dynamics of this model and bifurcation analysis of the key element of the CPG circuit, we conclude:

1. There are two limit cycles in the phase space of the model: an anti-phase limit cycle with period T which corresponds to swimming activity, and an in-phase limit cycle with period T/2 which corresponds to synchrony (see Figure 1).

2. The anti-phase (swimming) cycle is stable and robust with a large basin of attraction. The in-phase (synchrony) cycle can be initiated from swimming regime by mid-cycle stimulation of dIN neurons, but is generally unstable or has a very small basin of attraction.

3. The in-phase cycle is near a fold of limit cycles bifurcation. For a minority of generated connections the stable in-phase limit cycle exists. However, for most connections this stable cycle does not exist, although its “ghost” is still visible: after several synchronous cycles the system returns to swimming oscillations.

4. The stability of synchrony is extremely sensitive to the synaptic/conductance delays between dINs and cINs, with longer delays stabilizing synchrony.

5. It is unclear whether synchrony has a functional purpose. If it does, we hypothesize that in this early developmental stage the system may be near to the critical bifurcation point in order to provide flexibility in locomotion control. As the tadpole develops, longer synaptic delays may act to stabilize the synchronous firing pattern.
